# Transorbital penetrating intracranial injury involving bilateral frontal lobes with evisceration of right eye: A case report

**DOI:** 10.1002/ccr3.9018

**Published:** 2024-05-31

**Authors:** Abdul Rehman Siddiqui, Kaiser Kariem, Mohsin Fayaz, Gianluca Scalia, Bipin Chaurasia

**Affiliations:** ^1^ Department of Neurosurgery Super Specialty Hospital GMC, Shireen Bagh Srinagar Srinagar India; ^2^ Department of Neurosurgery Sher‐i‐Kashmir Institute of Medical Sciences Srinagar India; ^3^ Neurosurgery Unit, Department of Head and Neck Surgery Garibaldi Hospital Catania Italy; ^4^ Department of Neurosurgery Neurosurgery Clinic Birgunj Nepal

**Keywords:** computed tomography, duraplasty, eye, foreign body, injury, orbit

## Abstract

**Key Clinical Message:**

Timely diagnosis, multidisciplinary surgical intervention, and appropriate imaging are crucial in managing transorbital‐penetrating intracranial injuries (TOPIs), minimizing morbidity, and optimizing patient outcomes.

**Abstract:**

Transorbital‐penetrating intracranial injuries (TOPIs) are rare occurrences with potential for severe neurological complications and high mortality rates. Prompt diagnosis and management are essential to mitigate adverse outcomes. Understanding injury patterns and employing appropriate imaging modalities are crucial for effective surgical planning and patient care. We present a case of a 22‐year‐old male mechanic who sustained a TOPI involving bilateral frontal lobes with evisceration of the right eye following a workplace accident with a metal cutter. Upon arrival at the emergency department, the patient exhibited vision loss in the right eye, proptosis, and a dilated pupil. Imaging studies revealed the trajectory of a metal arrow through the right orbital roof, necessitating surgical intervention. A multidisciplinary team performed bifrontal craniectomy with duroplasty to remove the foreign body and address associated injuries. Postoperatively, the patient received broad‐spectrum antibiotics and anticonvulsants, leading to full recovery and discharge on postoperative day 10. TOPIs present unique challenges due to their rarity and potential for devastating consequences. Our case highlights the importance of timely diagnosis, meticulous surgical planning, and multidisciplinary collaboration in achieving favorable outcomes. Radiological imaging plays a crucial role in guiding treatment decisions and optimizing patient care. This report underscores the significance of early surgical intervention, antimicrobial therapy, and prophylactic measures in reducing morbidity and mortality associated with TOPIs.

## 1 INTRODUCTION

Accidental penetrating injuries of the brain are relatively uncommon.^1^ Although transorbital‐penetrating intracranial injury (TOPI) is rare, its potential for causing severe brain damage is associated with high mortality rates.^2^, ^3^ This injury, constituting 4.5% of all orbital abnormalities, represents only 0.04% of all head injuries, with penetrating head trauma incidents occurring in approximately 24% of adults and 45% of children.^4^ Despite its infrequency, TOPI can lead to significant damage to orbital and brain structures and may even result in death if not promptly treated.^5^, ^6^ This unusual injury is often accompanied by intracranial complications such as brain abscess, meningitis, cerebrospinal fluid (CSF) leakage, hemorrhage, neurological deficits, and mortality.^6^, ^7^ Vascular complications following TOPI are reported with a prevalence as high as 50%, and these can pose life‐threatening risks.^8^ The mortality rate for TOPIs is approximately 33% with timely surgical treatment but increases to 53% when surgery is delayed.^7^, ^9^ Surgical intervention is the primary treatment strategy for TOPIs, with indications including retained foreign bodies, CSF leakage, fracture displacement, intracranial hemorrhage, and vascular injury. Surgical approaches to remove orbital cranial foreign bodies can be categorized into two types based on location: extra‐orbital, such as transcranial, or transorbital approaches.^10^ This study presents an uncommon case of a male patient who experienced a TOPI, following the Surgical Case Report (SCARE) 2018 guidelines.^11^

## 2 CASE HISTORY/EXAMINATION/PRESENTATION

A 22‐year‐old male presented to our emergency department with vision loss in his right eye following an accident at his workplace. The patient, a mechanic, was using a machine cutter when a projectile approximately 2 cm in size struck his right eye, penetrating the frontal bone.

Upon arrival, the patient's vital signs were within normal limits, and he was alert. Primary and secondary surveys did not reveal additional injuries, and there was no significant medical history or history of drug, tobacco, or alcohol abuse. The patient reported a headache but no nausea, vomiting, or convulsions. Physical examination showed normal body temperature, a Glasgow Coma Scale score of 15, clear consciousness and speech, and normal muscular strength and tension in the limbs. Bilateral Babinski signs were not present. Preoperative tests, including blood analyses and biochemical parameters, were normal.

**FIGURE 1:**
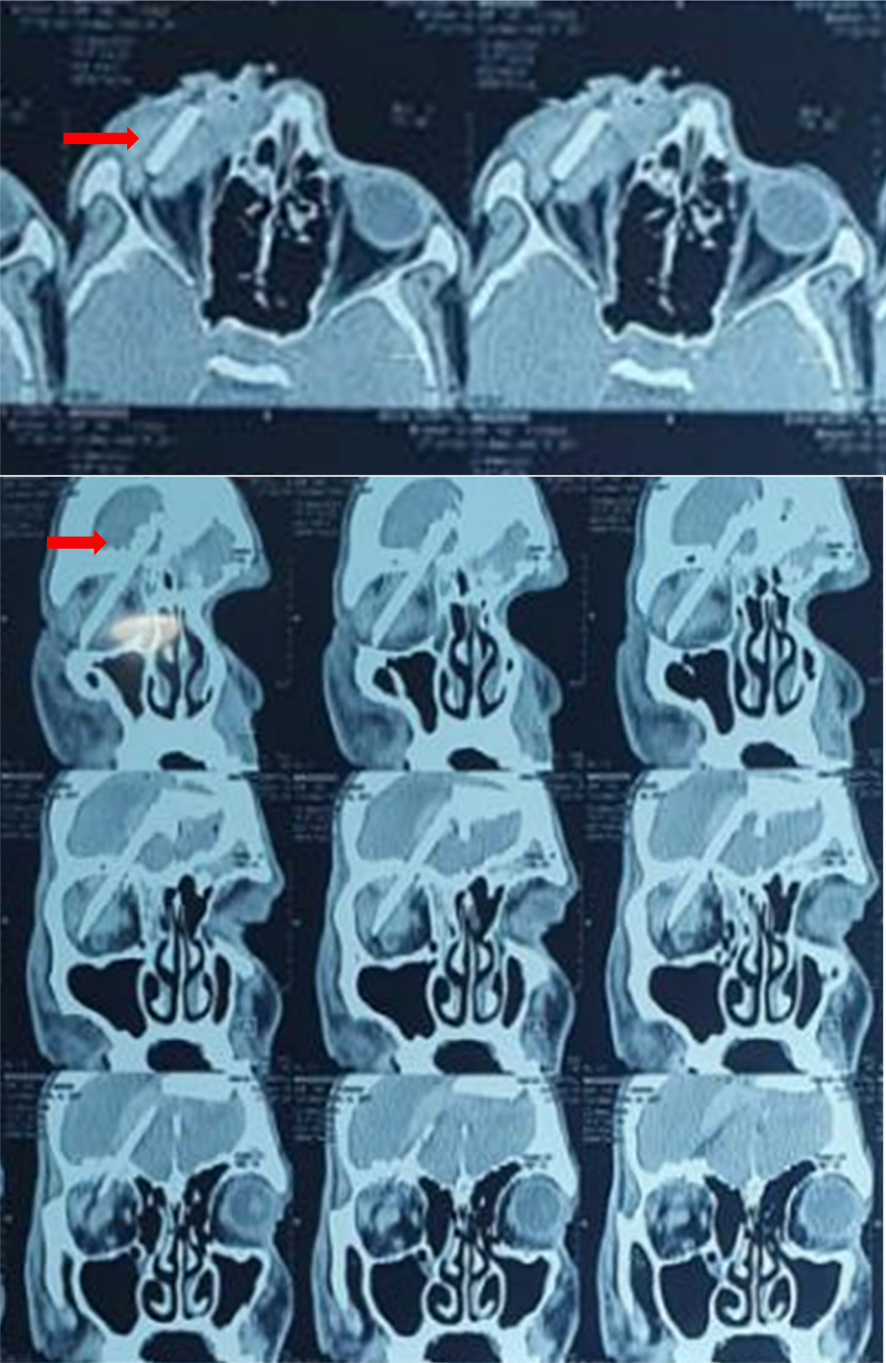
Non‐contrast head CT scan of the brain showed the trajectory of the metal arrow posteriorly through the right orbital roof (red arrows).

Local examination revealed a perforating injury at the entry point of the foreign body below the right infraorbital margin, causing proptosis of the right eyeball and bleeding. The right pupil was dilated and not reacting to light, with no eye movement. Additionally, a lacerated wound of approximately 5 cm was observed along the right frontal region. A non‐contrast CT scan of the brain showed the trajectory of the metal arrow posteriorly through the right orbital roof (Figure 1).

## 3 METHODS (DIFFERENTIAL DIAGNOSIS, INVESTIGATIONS, AND TREATMENT)

**FIGURE 2:**
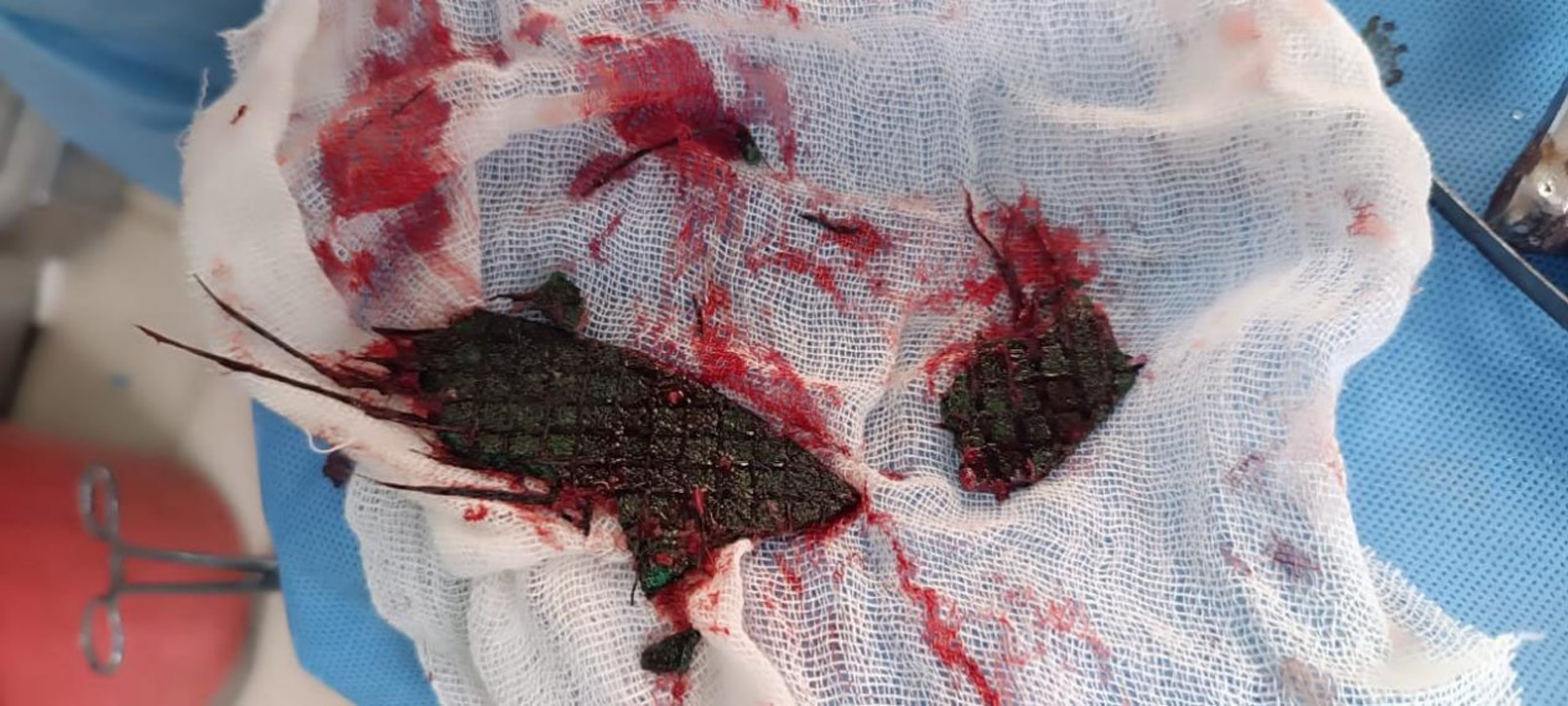
Intraoperative image of removed foreign body.

As there was no evidence of vascular injury, it was deemed safe to remove the foreign body via bifrontal craniectomy with duroplasty. A multidisciplinary approach involving a neurosurgeon and an ophthalmologist was employed. The patient underwent surgery 2 h after arrival in the emergency department. The foreign body was removed through the penetration wound, followed by evisceration of the eyeball (Figure 2). Minimal brain tissue protruded from the entry wound without CSF leakage. Intraoperative findings revealed a metallic foreign body penetrating through the right eye into the right frontal and left frontal lobes, damaging the dura mater. Another foreign body was found penetrating the right parietal lobe and superior sagittal sinus. Additionally, the right globe was damaged, and the right frontal bone and sinus were fractured. The wound was irrigated with a standard saline solution containing antibiotics to remove debris and control bleeding.

## 4 RESULTS (OUTCOME AND FOLLOW‐UP)

Postoperatively, the patient was admitted to the critical care unit, where broad‐spectrum antibiotics and anticonvulsant drugs were administered for 7 days. The patient fully recovered and was discharged on postoperative day 10. Long‐term follow‐up evaluations, including assessment of the surgical wound and the patient's general condition, were satisfactory. A CT scan of the brain with and without contrast revealed slight ischemia in the right frontal lobe and a globe rupture in the right eye. A slight ischemia along the foreign‐body track in the right frontal lobe and evisceration of the right eye were also shown. The patient's vision in the left eye remained normal, with no signs of sympathetic ophthalmia.

## 5 DISCUSSION

Transorbital‐penetrating injuries of the skull and brain are rare occurrences, yet they can lead to significant ophthalmic and neurological complications.^6^ The presentation of a transorbital‐penetrating intracranial injury varies depending on factors such as the size, orientation, type of object, and depth of penetration. Given the anatomical structure of the orbit as a rectangular pyramid, foreign bodies can penetrate through various edges, including the superior, inferior, lateral, or medial edges.^3^ The superior orbital fissure, situated at the apex, serves as a passage for cranial nerves such as III, IV, and VI into the middle cranial fossa.^10^ Foreign bodies longer than 5 cm can potentially penetrate the cranial cavity through the orbital cavity.^12^ In our case, the pathogenic foreign body exceeded 5 cm in length, penetrating the brain.

Foreign bodies can reach the brain via three main routes: the optic canal, the superior orbital fissure, and the orbital roof.^13^ The orbital roof, due to its fragile bone structure and thinner parts, is the most common route for penetration, allowing foreign bodies to reach the meninges, brain parenchyma, and vascular structures, often resulting in damage to the frontal lobe.^10^ Patterns of transorbital intracranial injury have been categorized into distinct zones, with our case falling into Zone 3C, the inferior medial region of the right orbit.^14^ Understanding these injury patterns is crucial for guiding management and surgical approaches, as well as anticipating potential intracranial complications associated with foreign‐body penetration.^14^, ^15^

In our case, imaging played a critical role in diagnosis and surgical planning. Ocular ultrasound revealed a discontinuity of the inferior wall of the eyeball, while plain skull radiography confirmed the penetration of the metal arrow through the right orbital roof and into the right parietal lobe. However, plain skull radiographs alone may have limitations in detecting certain deformities or foreign bodies, highlighting the importance of additional imaging modalities such as CT scans.^14^

Non‐contrast CT is considered the primary imaging modality for initial radiological assessment in cases of TOPIs.^5^, ^10^ CT scans can identify remaining foreign bodies, assess lesion extension, localize foreign bodies and bone fragments, detect hematoma, determine the penetration pathway, and provide valuable information for surgical planning.^16^ In our case, both standard CT and 3D CT scans provided detailed visualization of the bony pathology, foreign body trajectory, and associated abnormalities, aiding in surgical decision‐making.

Additionally, CT angiography (CTA) can be valuable for investigating cerebrovascular injuries following intracranial penetrating trauma. CTA is effective in detecting traumatic intracranial aneurysms, dissections, occlusions, or revealing the location of hematomas.^7^, ^18^, ^19^, ^20^ In our case, CTA ruled out discernible injury to the surrounding middle cerebral artery branches. Treatment of TOPIs aims to prevent infections and mitigate neurological sequelae. Surgical intervention is often necessary for removing foreign bodies, managing intracranial hematomas, displaced bone fractures, and addressing dural defects or vascular injuries.^18^ The choice of surgical approach, whether transorbital or transcranial, depends on factors such as the location of the foreign body and the presence of a vascular injury.^12^ Early surgical intervention is crucial, with the recommendation being surgery within 12 h to balance the risks of bleeding and infection.^17^, ^22^ Postoperatively, broad‐spectrum antibiotics are administered to prevent infections, which are among the most fatal complications of TOPIs. Retained foreign bodies and bone fragments in the wound track pose a significant risk of infection.^21^, ^24^ Antiepileptic drugs may also be administered prophylactically to reduce the risk of post‐traumatic epilepsy, a common complication following TOPIs.^20^, ^23^ In conclusion, although TOPIs are uncommon, they can lead to serious orbital and brain injuries, emphasizing the importance of prompt diagnosis and treatment. Understanding the patterns of injury and utilizing appropriate imaging modalities are crucial for guiding surgical management and optimizing patient outcomes. Early surgical intervention, combined with antimicrobial therapy and prophylactic measures, plays a key role in reducing morbidity and mortality associated with TOPIs.

## 6 CONCLUSION

In this case report, we detailed the presentation and management of a TOPI caused by a metal cutter accident. The outcome of TOPIs hinges on factors such as the trajectory of the penetration, extent of neurovascular injury, and neurological status of the patient. Our findings underscore the critical role of radiological imaging in diagnosing TOPIs and guiding surgical interventions. Moreover, a multidisciplinary approach involving neurosurgeons, ophthalmologists, and other specialists is vital for optimizing patient outcomes and reducing disability and mortality rates associated with this rare but potentially devastating injury.

## AUTHOR CONTRIBUTIONS

**Abdul Rehman Siddiqui:** Conceptualization; data curation; formal analysis. **Kaiser Kariem:** Conceptualization; data curation; formal analysis. **Mohsin Fayaz:** Conceptualization; data curation; formal analysis. **Gianluca Scalia:** Supervision; validation; visualization; writing – original draft; writing – review and editing. **Bipin Chaurasia:** Supervision; validation; visualization.

## FUNDING INFORMATION

This manuscript did not receive any funds.

## CONFLICT OF INTEREST STATEMENT

None.

## ETHICS STATEMENT

None.

## CONSENT

Written informed consent was obtained from the patient to publish this report in accordance with the journal's patient consent policy.

## Data Availability

Data sharing not applicable—no new data generated, or the article describes entirely theoretical research.
